# Chemical Characterization and DNA Fingerprinting of *Griffonia simplicifolia* Baill.

**DOI:** 10.3390/molecules24061032

**Published:** 2019-03-15

**Authors:** Ivano Vigliante, Giuseppe Mannino, Massimo E. Maffei

**Affiliations:** Plant Physiology Unit, Department of Life Sciences and Systems Biology, University of Turin, Via Quarello 15/a, 10135 Turin, Italy; ivano.vigliante@unito.it (I.V.); giuseppe.mannino@unito.it (G.M.)

**Keywords:** *Griffonia simplicifolia*, Caesalpiniaceae, 5-hydroxy-l-tryptophan (5-HTP), fatty acids, PCR–RFLP, internal transcribed spacer (ITS), DNA fingerprinting

## Abstract

Background: *Griffonia simplicifolia* Baill. (Caesalpiniaceae) is a medicinal plant whose seeds are widely used in traditional medicine for their high content of 5-hydroxy-l-tryptophan (5-HTP), a direct precursor and enhancer of the activity of the brain hormone serotonin (5-HT). The plant extracts are used in dietary supplements aimed to alleviate serotonin-related disorders. Methods: In order to characterize the chemical components of *G. simplicifolia* seeds and their identity, we used a combined methodology by using HPLC-DAD-ESI-MS/MS for the qualitative and quantitative determination of the *N*-containing compounds, GC-FID and GC-MS for the characterization of the major fatty acids, and DNA fingerprinting based on PCR–RFLP for the unequivocal identification of the plant. Results: 5-HTP was the most representative compound, followed by lower percentages of the β-carboline alkaloid derivative griffonine and other alkaloids. Fatty acids were dominated by the unsaturated fatty acids linoleic acid and oleic acid, followed by the saturated fatty acids stearic and palmitic acids. PCR analysis of the internal transcribed spacer amplified sequence showed a major band at about 758 bp, whereas the PCR–RFLP analysis of this sequence using three different restriction enzymes (*MspI*, *HhaI*, and *HaeIII*) generated a specific fingerprinting useful for the plant identification. Conclusions: The combined chemical and molecular analysis of *G. simplicifolia* provided an interesting integrated approach for the unequivocal identification of commercial *G. simplicifolia* seeds.

## 1. Introduction

The legume plant *Griffonia simplicifolia* Baill. (Caesalpiniaceae) (also known with the alternate incorrect name *Bandeiraea simplicifolia*) is a perennial woody shrub which grows in the tropical rain forest of West and Central Africa, with sites of cultivation present in Ghana, Ivory Coast, and Togo. The seed chemical constituents, including lectins, 5-hydroxy-l-tryptophan (5-HTP), and fatty acids have been studied intensively since 1960 [1,2], whereas leaves may contain lectin II—a legume lectin with GlcNAc binding specificity resulting in insecticidal activity [3].

Seed extracts from *G. simplicifolia* are rich in 5-HTP [4]—a direct precursor and enhancer of the activity of the brain hormone serotonin (5-hydroxytryptamine, 5-HT). After entering the central nervous system, 5-HTP is converted to 5-HT by the enzyme tryptophan decarboxylase [5]. The administration of 5-HTP to animals increases 5-HT levels in the central nervous system (CNS) [6]; however, in humans 5-HTP stimulates 5-HT receptors in the CNS only after conversion to 5-HT [7]. A medical food formulation comprised of *G. simplicifolia* containing high concentrations of 5-HTP is thought to be effective for serotonin-related disorders [8,9], including depression [10] and young subjects with high levels of romantic stress [11]. Further uses of *G. simplicifolia* seed extracts include the treatment of insomnia, migraine, headache, and the regulation of appetite leading to weight reduction in obese patients, as well as the regulation of mood, memory, and many other functions [12,13]. Other constituents of this plant are the β-carboline alkaloid derivative griffonine and other alkaloids, some of which show potential in vitro activity against HepG2 cells [14].

The UV absorption spectra of 5-HTP, tryptamine, tryptophan, and 5-hydroxytryptamine are very similar, with strong absorption between 230 and 300 nm, which makes UV spectrophotometric assay of 5-HTP in seed material almost impossible [15]. Therefore, HPLC methods coupled to mass spectrometry have been developed [15,16,17]. Moreover, the regioselective hydroxylation of tryptophan via chemical approaches is not easy, and this is why *G. simplicifolia* seeds are used for the extraction and the commercial production of 5-HTP. The recent development of metabolic engineering and protein engineering in combination with fundamental genetics and biochemistry has allowed the production of 5-HTP from tryptophan in heterologous systems [18,19]. A common sophistication of *G. simplicifolia* is the addition of 5-HTP derived from transgenic bacteria to the plant extract, in order to increase the 5-HTP content. However, the bacterial transformation may lead to tryptophan by-products (e.g., peak E as 1,1′-ethylidenebis(l-tryptophan) and peak UV-5 as 3-(phenylamino)alanine) that have been associated to diseases such as eosinophilia–myalgia syndrome [20,21,22].

Existing assay methods for the authentication of *G. simplicifolia* seeds are laborious, time-consuming, and sensitive to interference from co-occurring materials. In order to ensure reliable supplies of appropriate *G. simplicifolia* seed material, manufacturers require an efficient, dependable, and relatively rapid method for assessing the quality of seeds before export. Along with mass spectrometry-based analytical methods, the biomolecular characterization of food and medicinal plants offers a potent tool for their unequivocal identification [23]. Among techniques, internal transcribed spacer (ITS) is widely used in plant molecular systematics at the generic and species levels because of its potentially high resolution of inter- and intraspecific relationships [24].

The aim of this study was to combine the seed chemical composition with DNA fingerprinting of commercial *G. simplicifolia* seeds. Chemical analyses included that of fatty acids, the characterization of 5-HTP and other tryptophan-derived compounds, the β-carboline alkaloid derivative griffonine and other alkaloids, as well as the search for the so-called peaks E and UV-5. To provide reliable DNA fingerprinting, DNA restriction fragment length polymorphism (PCR–RFLP) analysis was performed on the *G. simplicifolia* ITS. To our knowledge, there are no data on ITS characterization and on the combined use of ITS and chemical data for the chemical and molecular characterization of *G. simplicifolia*. The combination of chemical and molecular data provided an interesting integrated approach for the unequivocal identification of commercial *G. simplicifolia* seeds.

## 2. Results and Discussion

### 2.1. 5-HTP Is the Major Nitrogen-Containing Compound of *G. simplicifolia*

The seeds of *G. simplicifolia* showed a 9.18% (±0.18) weight loss and a 3.66% (±0.20) ashes content. The total amount of *N*-containing compounds was about 18% on a dry weight basis (Table 1). The main compound of the soluble fraction was 5-HTP. The content of this compound was about 16% of the seed weight (156 mg g^−1^) (Table 1), which is consistent with the literature data reporting concentrations ranging from 10% to 20%, obtained through different analytical methods and different solvents [15,25,26]. These data are in contrast with claims of frequently found 5-HTP concentrations in *G. simplicifolia* seed extracts of 98% [26]. As shown in Table 1, these high percentages can be obtained if the relative percentage is calculated based only on the total identified compounds. Another possibility for the high percentage found in *G. simplicifolia* extracts might be the sophistication with 5-HTP from genetically engineered bacterial fermentation, as frequently reported [27], or by filtration of culture media through a reverse osmosis membrane to remove chemicals with a molecular weight higher than 1000 [20]. One way to determine the quality of *G. simplicifolia* extracts is to evaluate the presence and content of their characteristic alkaloids [14]. The alkaloid hyrtioerectine B was present at about 7 mg g^−1^ on a seed dry weight basis (Table 1). This compound is also present in the marine sponge *Hyrtios erectus* and showed moderate cytotoxicity against HeLa cancer cells with an IC_50_ of 5.0 μg mL^−1^ [28] and was found to inhibit HepG2 cell lines with IC_50_ values of 9.6 μmol L^−1^ [14]. The 3-carboxy-6-hydroxy-β-carboline content was close to 3 mg g^−1^, whereas 5-hydroxytryptamine was present at lower concentrations (Table 1). These two compounds were found to be ineffective against HepG2 cell lines [14]. Minor contents were found for griffonine and hyrtiosulawesine. These two compounds were potentially active against the HepG2 cancer cell line in vitro [14]. Other minor compounds were 1H-indole-3-carboxylic acid and 5-hydroxy-3-(2-hydroxyethyl)indole (Table 1).

**Figure 1:**
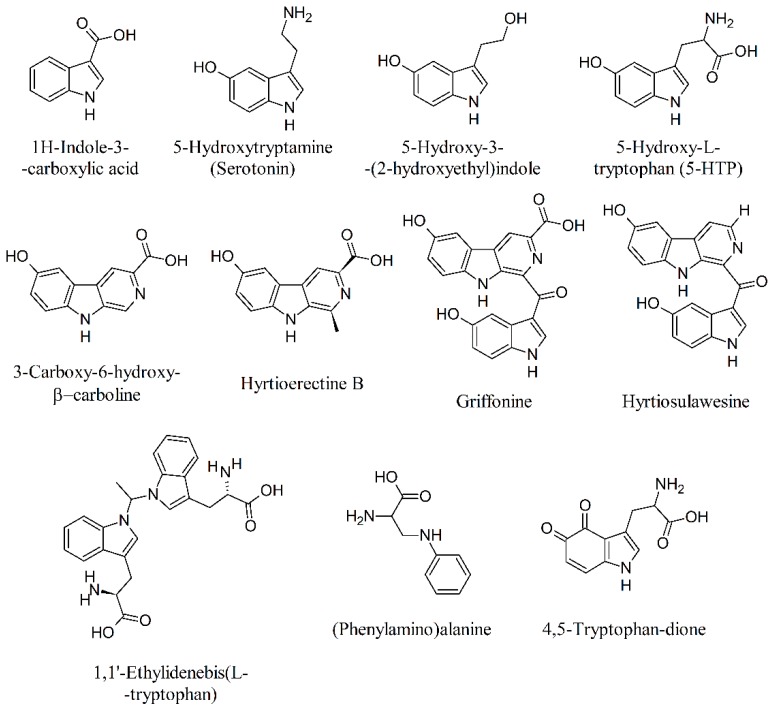
Structures of different isolated N-containing compounds from *G. simplicifolia*.

The search for the neurotoxic compounds 1,1′-ethylidenebis(l-tryptophan) (peak E) and 3-(phenylamino)alanine (peak UV-5) was ineffective, demonstrating that these two compounds are typical markers of engineered bacterial fermentation [20,29]. On the other hand, peak X_1_ (4,5-tryptophan-dione)—the oxidation product of 5-HTP [27]—was present at a concentration of about 12 mg g^−1^ on a dry weight basis (Table 1). By considering that the suggested recommended doses are around 10–50 mg of 5-HTP/day [30], this would provide about 0.7–3.7 mg 4,5-tryptophan-dione. Although the direct toxicity of this compound is unknown at present, possible reactions with reduced glutathione cannot be excluded [27]. Figure 1 shows the chemical structure of the compounds listed in Table 1.

The data reported here, and a review of the literature [14,15,25,26], suggest that an original and natural *G. simplicifolia* extract should contain a 5-HTP:alkaloid ratio of about 6–7:1. Higher ratios might indicate a different 5-HTP origin or the use of fractionation methods during extraction.

As a proof of concept, we analyzed a commercial sample of a *G. simplicifolia* extract claiming a 95% 5-HTP content. After the chemical characterization, we found that the real content of 5-HTP was about 65%, that the ratio between 5-HTP and other alkaloids was 14:1, and the presence of peak E was about 6 mg g^−1^, which indicates a possible bacterial fermentation process (see Supplementary data S2).

### 2.2. *Griffonia simplicifolia* Seeds Show a High Content of Unsaturated Fatty Acids

The lipid composition of *G. simplicifolia* seeds was characterized by the presence of unsaturated and saturated fatty acids, as is typical for a south thermophilic plant. The total content of the major fatty acids was about 165 (±11) mg g^−1^ on a dry weight basis (Table 2). Among unsaturated fatty acids, linoleic acid (55%) and oleic acid (12%) were the most abundant compounds, whereas the remaining fatty acids were represented by saturated fatty acids, with stearic acid (20%) and palmitic acid (11%) being the most abundant (Table 2). The results reported here are in line with literature data, confirming the prevalence of unsaturated fatty acids in *G. simplicifolia* seeds [1].

### 2.3. DNA Fingerprinting of *Griffonia simplicifolia* Using PCR–RFLP Analysis

**Figure 2:**
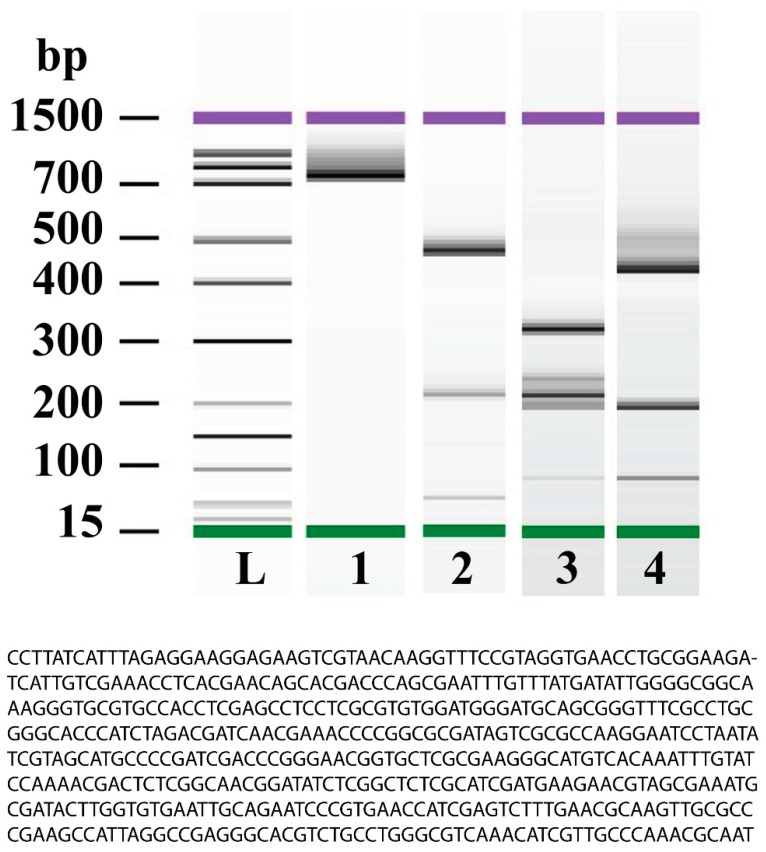
Electrophoresis representation of the Agilent Bioanalyzer 2100 gel-electropherograms of PCR products generated by primers flanking the internal transcribed spacer (ITS) region of ITS-1 and ITS-4 and PCR–restriction fragment length polymorphism (RFLP) products from *G. simplicifolia*. Lane 1, PCR product of the spacer region produced a band of about 758 bp; Lane 2, PCR–RFLP analysis using MspI *G. simplicifolia* digested PCR products gave three bands of about 471, 312, and 57 bp; Lane 3, PCR–RFLP analysis using HhaI *G. simplicifolia* digested PCR products produced five bands of about 319, 240, 215, 198, and 86 bp; Lane 4, PCR–RFLP analysis using HaeIII *G. simplicifolia* digested PCR products generated three bands of about 431, 198, and 86 bp. L = bp markers. The green bar indicates the lower marker (15 bp), whereas the violet band indicates the upper marker (1500 bp). See Supplementary data S1 for more information. The figure also shows the nucleotide sequences of the ITS gene spacer region of *G. simplicifolia*.

In order to provide molecular fingerprinting of *G. simplicifolia* seeds, ITS-1 coupled with ITS-4 was used for PCR amplification. Figure 2 shows the nucleotide sequence of the ITS region of *G. simplicifolia*. In general, the ITS-amplified sequence was about 758 bp long (NCBI GenBank Accession No MH707248) (Figure 2 lane 1). In order to provide a better-defined DNA fingerprint, a PCR–RFLP method was applied. Three different restriction enzymes (MspI, HhaI, and HaeIII) were used to selectively cleave the resulting amplicon. Digestion of the PCR product with MspI generated three fragments: one major fragment of about 471 bp, and two minor fragments of about 57 and 213 bp (Figure 2 lane 2). An HhaI site could also be identified in the ITS region, generating five distinct fragments: the major fragment gave a band of about 319 bp, followed by a band of about 215 bp. Two minor bands were found at about 198 and 240 bp, and there was a faint band at 86 bp (Figure 2 lane 3). Finally, digestion with HaeIII generated three bands: a major band of about 431 bp, followed by two bands of minor concentration of about 198 and 86 bp (Figure 2 lane 4). Supplementary data S1 provides the electropherograms and the concentration and molarity of both the ITS region and PCR–RFLP products. To our knowledge, this is the first report on the DNA fingerprinting of *G. simplicifolia*. 

## 3. Materials and Methods 

### 3.1. Plant Material

Seeds of *Griffonia simplicifolia* Baill. (Caesalpiniaceae) were kindly provided by Demar Srl Cesena (FC), Italy, batch 171/10/17/722, and were collected from plants originating from Ivory Coast (North-West Africa). Seeds were stored in the dark at 4 °C before extraction. At least three technical replicates were done for each lot of seeds. The dry weight of the seeds was obtained after placing the seeds overnight in an oven at 105 °C, whereas ashes were obtained by placing the seeds in a muffle at 600 °C for 6 h.

### 3.2. Extraction of 5-HTP and Other N-Containing Compounds 

For 5-HTP and alkaloid analysis, ground seeds of *G. simplicifolia* were extracted by maceration in an 1:10 *w*/*v* ethanol:water 70:20 *v*/*v* solution for 3 days in the dark at room temperature. Extraction provided a soluble and an insoluble fraction. The supernatant soluble fraction was recovered after centrifugation (10 min at 10,000× *g*, at 4 °C) and filtration through a Millex HV 0.45 μm filter (Millipore, Billerica, MA, USA). In order to guarantee an exhaustive extraction, pellets were re-extracted twice. After centrifugation and filtration, the supernatants were combined and stored at −80 °C until analysis.

Lipophilic extracts of *G. simplicifolia* seeds were obtained by Soxhlet extraction for 6 h with cyclohexane (1:10, *w*/*v*). After extraction, the solvent was removed with a nitrogen flow and the extract stored at −20 °C until analysis.

### 3.3. Identification and Quantification of 5-HTP and Other N-Containing Compounds 

The HPLC system consisted of an Agilent Technologies 1200 coupled to a DAD and a 6330 Series Ion Trap LC-MS System (Agilent Technologies, Santa Clara, CA, USA) equipped with an electrospray ionization (ESI) source. The chromatographic separation was carried out at constant flow rate (0.2 mL min^−1^). The column was a reverse-phase C18 Luna column (3.00 μm, 150 mm × 3.0 mm i.d., Phenomenex, Torrance, CA, USA) maintained at 25 °C by an Agilent 1100 HPLC G1316A Column Compartment. The binary solvent system for the analysis of *N*-containing compounds was MilliQ H_2_O acidified with 0.1% *v*/*v* formic acid (Solvent A) and acetonitrile acidified with 0.1% *v*/*v* formic acid (Solvent B). The samples were separated by an isocratic gradient of 97% A and 3% B. In order to clean the column from other interferences before the next injection, the concentration of B was slowly raised and maintained at 3% for 5 min. Sample injection volume was set to 5 μL. The UV–VIS spectra were recorded between 220 and 500 nm and the chromatographic profiles were registered at 230 and 270 nm. Tandem mass spectrometry analyses were performed by operating in positive mode. The nitrogen flow rate was set at 5.0 mL min^−1^ and maintained at 325 °C, whereas the capillary voltage was set at 1.5 kV. Helium was used as a collision gas. Compound identification was carried out by comparison of the retention time and UV–VIS/MS spectra with those of authentic reference compounds or using literature data. Limit of detection (LOD) and limit of quantification (LOQ) for each compound was determined as previously described [31].

### 3.4. Identification and Quantification of Fatty Acids

Lipophilic extracts of *G. simplicifolia* seeds were esterified with boron tri-fluoride (10% *w*/*v* in methanol). An internal standard of 50 μg heptadecanoic acid (C17:0) was added. The fatty acid methyl esters (FAMEs) were obtained by acid catalysis according to Christie and Han [32] and were dehydrated with anhydrous MgSO_4_. FAME identification and quantification was performed by GC-MS (5975T, Agilent Technologies, USA) and by GC-FID (GC-2010 Plus, SHIMADZU, Kyoto, Japan), respectively, as previously described [33]. Compounds were identified through comparison of mass fragmentation spectra with reference NIST 98 spectra or by comparison of Kovats indexes and internal standard co-injection of pure standards (Sigma-Aldrich, St Louis, MO, USA). FAME quantification was obtained by internal standard. At least three technical replicates were run for each lot of *G. simplicifolia* seeds.

### 3.5. DNA Extraction, PCR Amplification, Subcloning, and Sequencing

Whole *G. simplicifolia* seeds were pulverized in liquid N_2_ using a mortar and pestle. Genomic DNA was extracted and quantified according to Capuzzo and Maffei [34]. Briefly, 20 ng of genomic DNA were used as a template for PCR amplification with specific primers for ITS-p5 (5’-CTTATCAYTTAGAGGAAGGAG-3’) and ITS-u4 (5’-RGTTTCTTTTCCTCCGCTTA-3’). PCR products were separated and purified as previously described [33]. Subcloning of the purified products was obtained by using the TOPO-TA Cloning Kit (Thermo Fisher Scientific, Waltham, MA, USA) followed by transformation in *Escherichia coli* sub-cloning DH5α Efficiency Competent Cells (Invitrogen, Paisley, UK). Colonies containing DNA inserts of the correct size were picked and grown overnight in 5 mL Luria-Bertani liquid medium. The mini-preparation of plasmid DNAs was carried out using NucleoSpin Plasmid Miniprep Kit (Macherey-Nagel, Düren, Germany). Plasmid DNAs were used as a template for sequencing (Macrogen, Wageningen, Holland). Both DNA strands were sequenced.

### 3.6. PCR–RFLP Analysis and DNA Fingerprinting

PCR products of the ITS gene were digested at 37 °C for 15 min with 10 U MspI, HhaI, or HaeIII (NEB, New England Biolabs, Ipswich, AM, USA) at 37 °C for 15 min. For each digestion reaction 1 μL was analyzed by capillary gel electrophoresis using the Agilent 2100 Bioanalyzer (Agilent Technologies) and the DNA 1000 LabChip Kit (Agilent Technologies) following the manufacturer’s instructions.

### 3.7. Statistical Analysis

Data are expressed as the mean of three technical replicates for each lot of seeds. ANOVA followed by Tukey–Kramer’s HSD post-hoc test (*p* < 0.05) was used to determine significant differences. All statistical analyses were performed by using the SYSTAT 10 software.

## 4. Conclusions

In conclusion, our data showed that natural *G. simplicifolia* seed extract was characterized by 5-HTP percentages that rarely exceeded 20% on a dry weight basis. Therefore, claims of 98% 5-HTP in *G. simplicifolia* seed extracts should be carefully considered. Moreover, natural *G. simplicifolia* extracts did not contain peaks E and UV-5, which are typical of transgenic bacterial fermentation used for the production of 5-HTP. We showed that a good indicator of *G. simplicifolia* natural extract is the ratio between 5-HTP and the other *N*-containing compounds. Our results also indicated that only analytical methods based on HPLC coupled to mass spectrometry allow the precise quantification and identification of *N*-containing compounds, and other methodologies (UV–VIS or sole HPLC) were insufficient to determine their identity and content. We also provided a molecular characterization of *G. simplicifolia* seeds by DNA fingerprinting using specific PCR–RFLP markers and our data provided a simple and unequivocal method for the plant identification. DNA fingerprinting offers the advantage of a fast and rather inexpensive method for the unequivocal identification of this plant species long before the chemical analysis is done. Moreover, being restricted to DNA, this analysis it is not affected by environmental factors that might affect the expression of those genes involved in 5-HTP production.

## Figures and Tables

**Table 1:** Chemical composition of nitrogen-containing compounds extracted from seeds of *Griffonia simplicifolia*.

Compound	MW	[M + H]^+^	*m*/*z*			mg g^−1^ d.wt.	SD	Relative Percentage
1H-indole-3-carboxylic acid	161.0	162.0	143.8	115.9		0.08	0.006	0.04
5-hydroxytryptamine	176.0	177.0	158.8	135.8	117.0	1.15	0.066	0.64
5-hydroxy-3-(2-hydroxyethyl)indole	177.0	178.0	159.8	132.9	115.0	0.27	0.006	0.15
5-hydroxy-l-tryptophan (5-HTP)	220.0	221.0	204.0	161.9		156.48	8.320	86.48
3-carboxy-6-hydroxy-β-carboline	228.0	229.0	210.0	183.0	101.1	2.69	0.120	1.49
hyrtioerectine B	246.0	247.0	229.9	203.9	174.0	6.75	0.320	3.73
griffonine	329.0	330.0	167.9			0.75	0.030	0.41
hyrtiosulawesine	343.0	344.0	228.9	200.9	182.9	0.9	0.030	0.50
1,1′-ethylidenebis(l-tryptophan)	434.0	435.0	231.0			n.d.	n.d.	n.d.
3-(phenylamino)alanine	180.0	181.0	106.0	88.0	70.0	n.d.	n.d.	n.d.
4,5-tryptophan-dione	234.0	235.0	217.0	162.0	181.0	11.87	0.92	6.56
TOTAL						181.10	16.10	100.00

SD: standard deviation; n.d.: not detectable (i.e., below the limit of detection).

**Table 2:** Fatty acid composition of *G. simplicifolia* seeds.

Fatty Acid	Kovat’s Index	mg/g	SD	Relative Percentage
palmitic acid	1942	18.22	0.93	11.06%
linoleic acid	2095	91.24	5.74	55.38%
oleic acid	2113	20.24	1.13	12.28%
stearic acid	2187	32.12	2.25	19.50%
arachidic acid	2359	10.39	0.65	6.31%
behenic acid	2400	4.65	0.32	2.82%
TOTAL		164.76	11.09	100.00%

## Supplementary Materials

The following are available online, Supplementary data S1: Electrophoresis assay details illustrating the gel electropherograms obtained to plot Figure 2; Supplementary data S2: Chemical characterization of a *Griffonia simplicifolia* extract claiming a 95% content of 5-HTP.
