# Supplementary material for: Chemical Characterization and DNA Fingerprinting of Griffonia simplicifolia Baill
Source: Molecules. 2019 Mar 15;24(6):1032. doi: 10.3390/molecules24061032 (PMC6472197; doi:10.3390/molecules24061032)
Supplement: Supplementary file 1 [file molecules-24-01032-s001.pdf]

SUPPLEMENTARY MATERIAL S1

# Electrophoresis Assay Details

## General Analysis Settings

Number of Available Sample and Ladder Wells (Max.) : 13 Minimum Visible Range [s] : 30
Maximum Visible Range [s] : 129
Start Analysis Time Range [s] : 30
End Analysis Time Range [s] : 128.95
Ladder Concentration [ng/µl] : 44
Uses Standard Area for Ladder Fragments
Lower Marker Concentration [ng/µl] : 4.2
Upper Marker Concentration [ng/µl] : 2.1
Used Upper Marker for Quantitation
Standard Curve Fit is Point to Point
Show Data Aligned to Lower and Upper Marker

## Integrator Settings

Integration Start Time [s] : 30
Integration End Time [s] : 128.95
Slope Threshold : 0.5
Height Threshold [FU] : 20
Area Threshold : 0.1
Width Threshold [s] : 0.5
Baseline Plateau [s] : 0.5

## Filter Settings

Filter Width [s] : 0.5
Polynomial Order : 4

| Ladder Peak Ladder | Size | Area |
|---|---|---|
| 1 | 15 | 25 |
| 2 | 25 | 26 |
| 3 | 50 | 34 |
| 4 | 100 | 41 |
| 5 | 150 | 45 |
| 6 | 200 | 52 |
| 7 | 300 | 63 |
| 8 | 400 | 76 |
| 9 | 500 | 83 |
| 10 | 700 | 88 |
| 11 | 850 | 86 |
| 12 | 1000 | 90 |
| 13 | 1500 | 52 |

| | | | |
|---|---|---|---|
| Assay Class: | DNA 1000 | Created: | 9/13/2018 11:36:39 AM |
| Data Path: | C:\...-13\2100 expert_DNA 1000_DE34903611_2018-09-13_11-36-39.xad | Modified: | 9/13/2018 12:18:35 PM |

## Electropherogram Summary

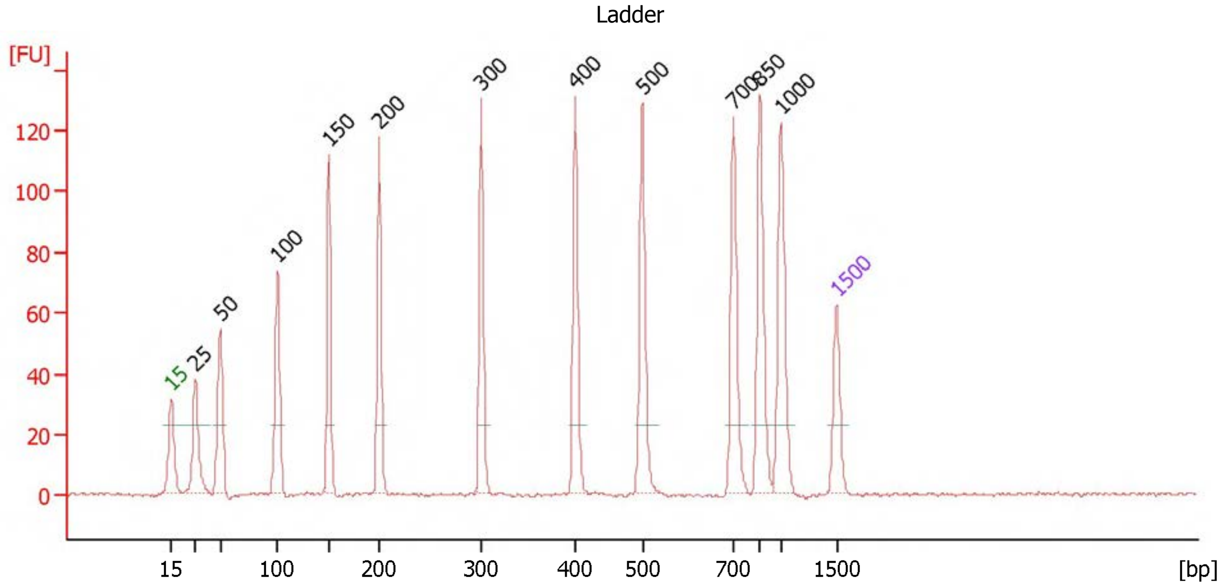


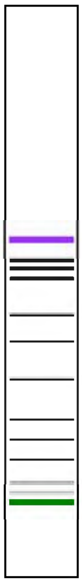

### Peak table for Ladder

| Peak | | Size [bp] | Conc. [ng/µl] | Molarity [nmol/l] | Observations |
|---|---|---|---|---|---|
| 1 | | 15 | 4.20 | 424.2 | Lower Marker |
| 2 | | 25 | 4.00 | 242.4 | Ladder Peak |
| 3 | | 50 | 4.00 | 121.2 | Ladder Peak |
| 4 | | 100 | 4.00 | 60.6 | Ladder Peak |
| 5 | | 150 | 4.00 | 40.4 | Ladder Peak |
| 6 | | 200 | 4.00 | 30.3 | Ladder Peak |
| 7 | | 300 | 4.00 | 20.2 | Ladder Peak |
| 8 | | 400 | 4.00 | 15.2 | Ladder Peak |
| 9 | | 500 | 4.00 | 12.1 | Ladder Peak |
| 10 | | 700 | 4.00 | 8.7 | Ladder Peak |
| 11 | | 850 | 4.00 | 7.1 | Ladder Peak |
| 12 | | 1,000 | 4.00 | 6.1 | Ladder Peak |
| 13 | | 1,500 | 2.10 | 2.1 | Upper Marker |

**Electropherogram Summary Continued ...**

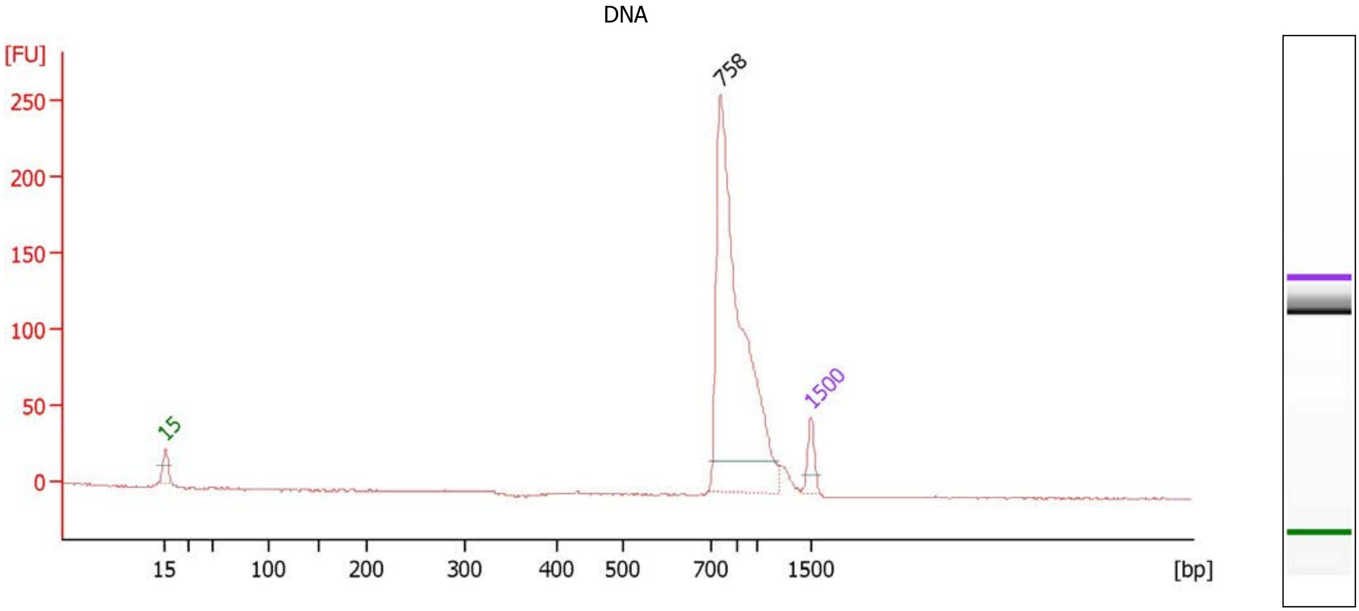


**Overall Results for sample 1 : DNA**

Number of peaks found: 1

**Peak table for sample 1 : DNA**

| Peak | | Size [bp] | Conc. [ng/µl] | Molarity [nmol/l] | Observations |
|---|---|---|---|---|---|
| 1 | ◀ | 15 | 4.20 | 424.2 | Lower Marker |
| 2 | | 758 | 43.61 | 87.2 | |
| 3 | ▶ | 1,500 | 2.10 | 2.1 | Upper Marker |

Assay Class: DNA 1000
Data Path: C:\...-13\2100 expert_DNA 1000_DE34903611_2018-09-13_11-36-39.xad

Created: 9/13/2018 11:36:39 AM
Modified: 9/13/2018 12:18:35 PM

**Electropherogram Summary Continued ...**

Msp I

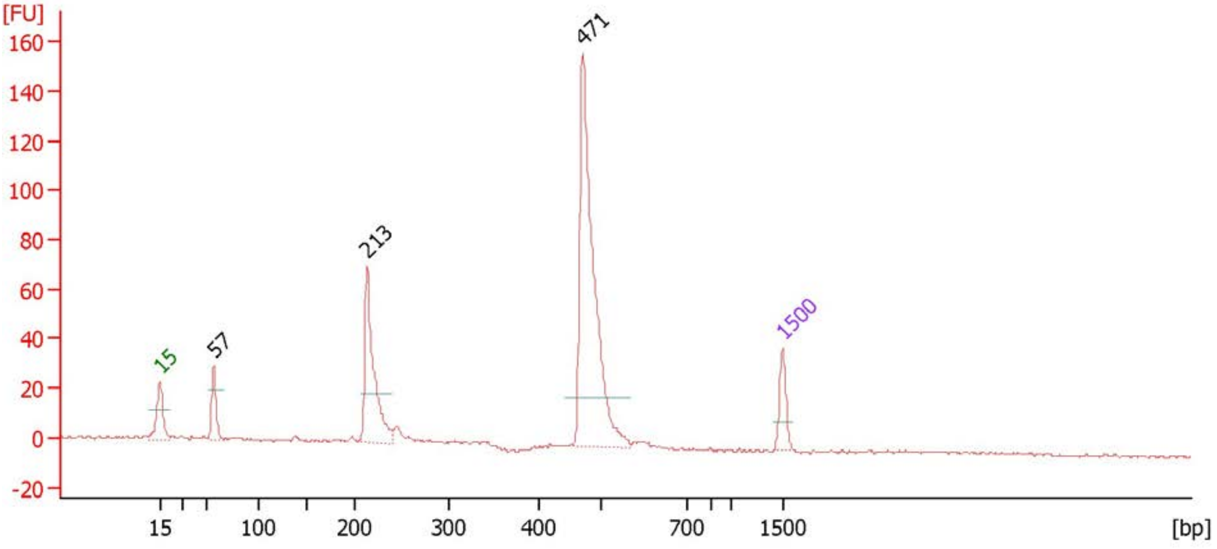


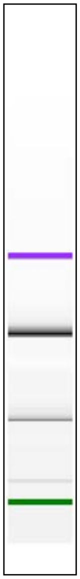

**Overall Results for sample 2 : Msp I**

Number of peaks found: 3

**Peak table for sample 2 : Msp I**

| Peak | | Size [bp] | Conc. [ng/µl] | Molarity [nmol/l] | Observations |
|---|---|---|---|---|---|
| 1 | ◀ | 15 | 4.20 | 424.2 | Lower Marker |
| 2 | | 57 | 2.94 | 77.8 | |
| 3 | | 213 | 9.19 | 65.3 | |
| 4 | | 471 | 20.65 | 66.4 | |
| 5 | ▶ | 1,500 | 2.10 | 2.1 | Upper Marker |

**Electropherogram Summary Continued ...**

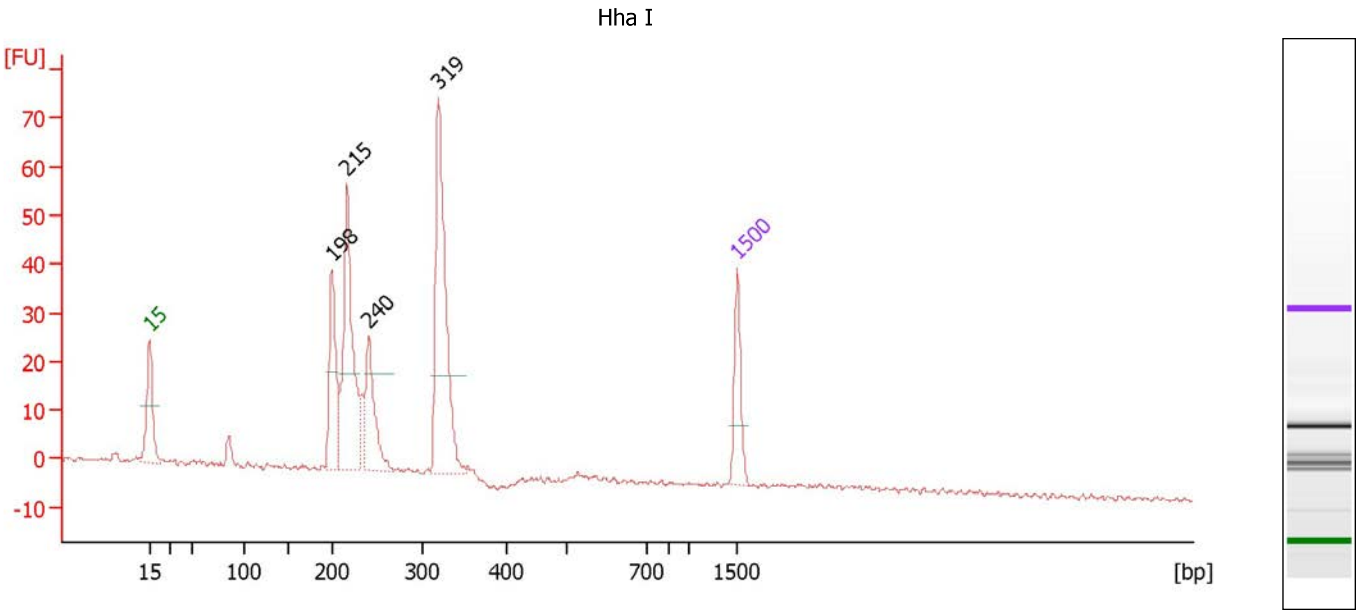


**Overall Results for sample 4 : Hha I**

Number of peaks found: 4

**Peak table for sample 4 : Hha I**

| Peak | | Size [bp] | Conc. [ng/µl] | Molarity [nmol/l] | Observations |
|---|---|---|---|---|---|
| 1 | ◀ | 15 | 4.20 | 424.2 | Lower Marker |
| 2 | | 198 | 3.95 | 30.2 | |
| 3 | | 215 | 7.52 | 53.0 | |
| 4 | | 240 | 3.32 | 21.0 | |
| 5 | | 319 | 8.72 | 41.5 | |
| 6 | ▶ | 1,500 | 2.10 | 2.1 | Upper Marker |

**Electropherogram Summary Continued ...**

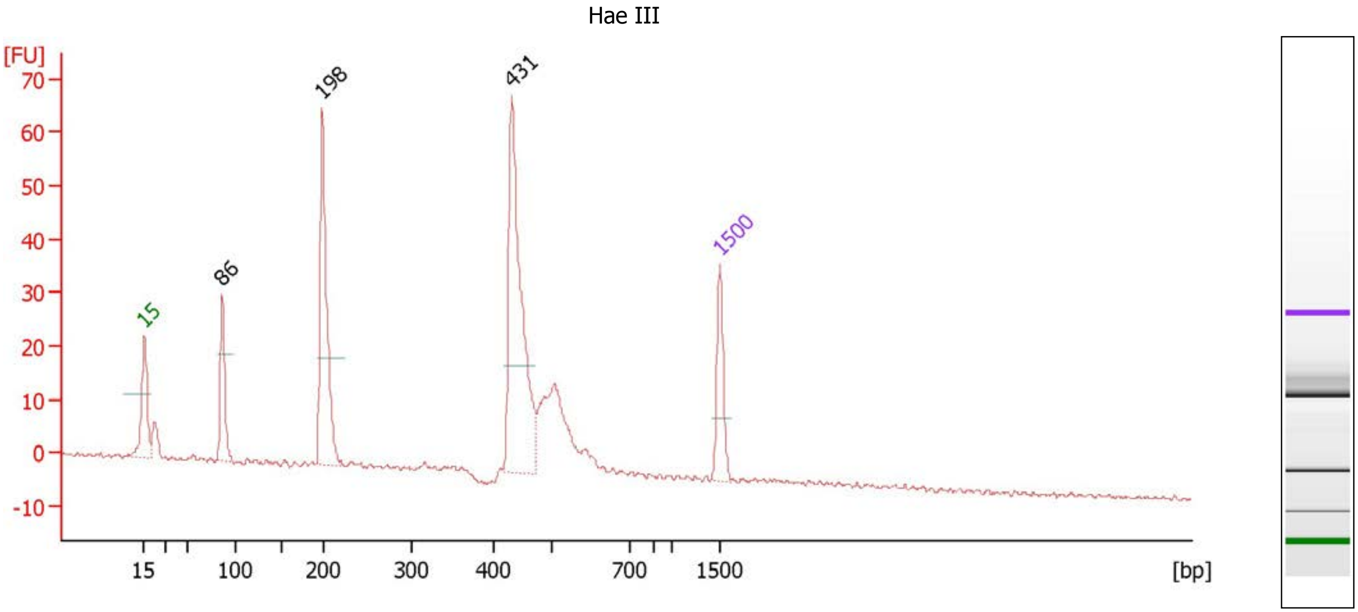


**Overall Results for sample 5 : Hae III**

Number of peaks found: 3

**Peak table for sample 5 : Hae III**

| Peak | | Size [bp] | Conc. [ng/µl] | Molarity [nmol/l] | Observations |
|---|---|---|---|---|---|
| 1 | ◀ | 15 | 4.20 | 424.2 | Lower Marker |
| 2 | | 86 | 2.96 | 52.1 | |
| 3 | | 198 | 6.75 | 51.7 | |
| 4 | | 431 | 9.05 | 31.8 | |
| 5 | ▶ | 1,500 | 2.10 | 2.1 | Upper Marker |

Assay Class: DNA 1000
Data Path: C:\...-13\2100 expert_DNA 1000_DE34903611_2018-09-13_11-36-39.xad

Created: 9/13/2018 11:36:39 AM
Modified: 9/13/2018 12:18:35 PM

## Curves

### Standard Curve

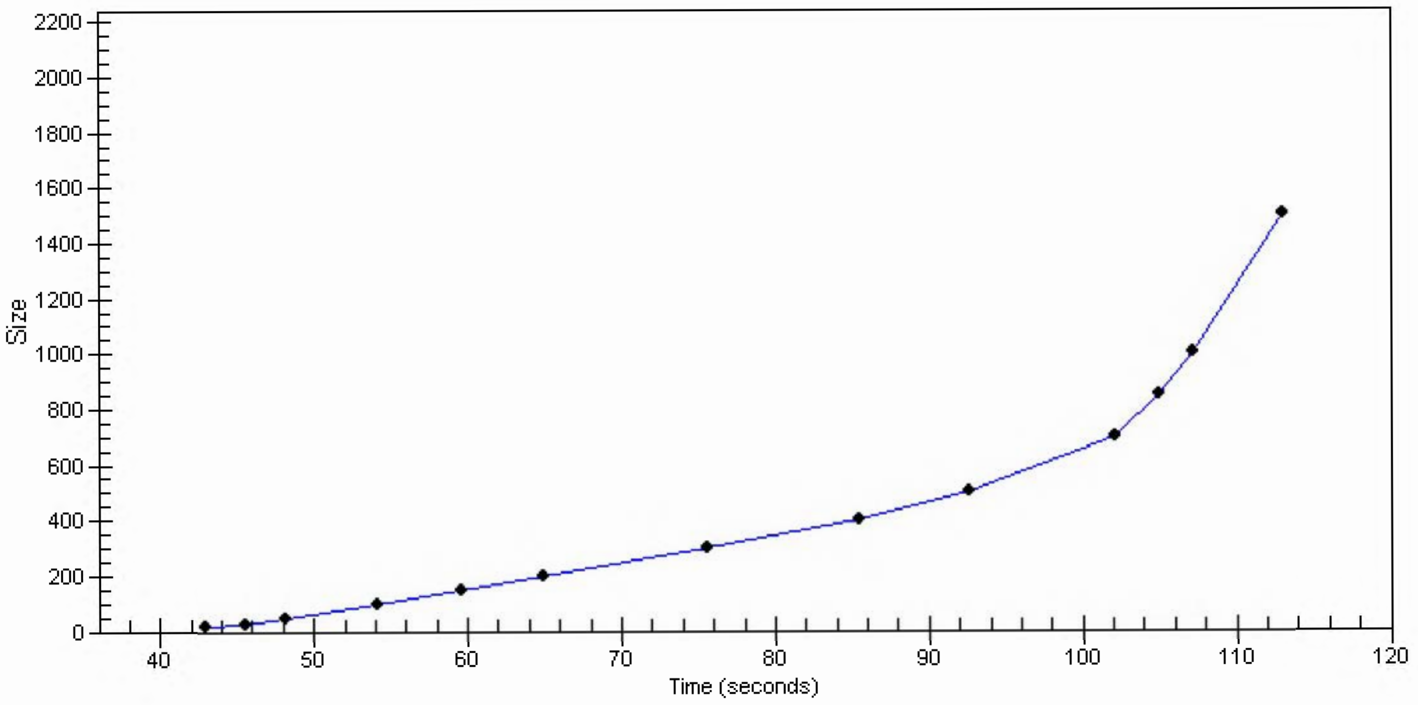

Assay Class: DNA 1000
Data Path: C:\...-13\2100 expert_DNA 1000_DE34903611_2018-09-13_11-36-39.xad

Created: 9/13/2018 11:36:39 AM
Modified: 9/13/2018 12:18:35 PM

## Run Logbook

| Description | Number | Source | Category | Sub Category | Time Stamp | Time Zone | User | Host |
|---|---|---|---|---|---|---|---|---|
| Run ended on port 2 (Number of wells acquired: 13) | | Instrument | Run | | 9/13/2018 12:18:29 PM | (GMT +02:00) W. Europe Standard Time | bioanalyser-user | LAB7-14 |
| Run started on port 2 (File: C:\Documents and Settings\bioanalyser-user\Desktop\bio-data\2018-09-13\2100 expert_DNA 1000_DE34903611_2018-09-13_11-36-39.xad) | | Instrument | Run | | 9/13/2018 11:36:46 AM | (GMT +02:00) W. Europe Standard Time | bioanalyser-user | LAB7-14 |
| Product Number : G2938C | | Instrument | Run | | 9/13/2018 11:36:46 AM | (GMT +02:00) W. Europe Standard Time | bioanalyser-user | LAB7-14 |
| Name : | | Instrument | Run | | 9/13/2018 11:36:46 AM | (GMT +02:00) W. Europe Standard Time | bioanalyser-user | LAB7-14 |
| Vendor : Agilent Technologies | | Instrument | Run | | 9/13/2018 11:36:46 AM | (GMT +02:00) W. Europe Standard Time | bioanalyser-user | LAB7-14 |
| Serial# : DE34903611 | | Instrument | Run | | 9/13/2018 11:36:46 AM | (GMT +02:00) W. Europe Standard Time | bioanalyser-user | LAB7-14 |
| Firmware : C.01.069 | | Instrument | Run | | 9/13/2018 11:36:46 AM | (GMT +02:00) W. Europe Standard Time | bioanalyser-user | LAB7-14 |
| Cartridge : Electrode | | Instrument | Run | | 9/13/2018 11:36:45 AM | (GMT +02:00) W. Europe Standard Time | bioanalyser-user | LAB7-14 |

# Supplementary Data S2: CHEMICAL CHARACTERIZATION OF A GRIFFONIA SIMPLICIFOLIA EXTRACT CLAIMING A 95% CONTENT OF 5-HTTP

| [C] | inj | Area |
|---|---|---|
| mg/mL | µL | Int. |
| 0,5 | 1 | 4,82E+09 |
| 0,25 | 1 | 2,62E+09 |
| 0,125 | 1 | 1,67E+09 |
| 0,0625 | 1 | 9,08E+08 |
| 0,03125 | 1 | 4,66E+08 |
| 0,015625 | 1 | 2,65E+08 |
| 0,007813 | 1 | 1,14E+08 |

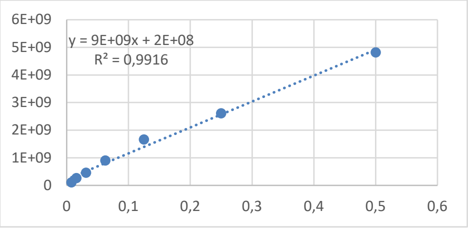


| m | q |
|---|---|
| 1E+10 | 0 |
| 4,13E+08 | #N/D |
| 0,989983 | 2,38E+08 |
| 592,9599 | 6 |
| 3,36E+19 | 3,4E+17 |
| #N/D | #N/D |

| RT | Compound | MW | $[M+H]^+$ | m/z | | |
|---|---|---|---|---|---|---|
| 35,10 | 1H-indole-3-carboxylic acid | 161,0 | 162,0 | 143,8 | 115,9 | |
| 38,10 | 5-hydroxytryptamine | 176,0 | 177,0 | 158,8 | 135,8 | 117,0 |
| 4,35 | 5-hydroxy-3-(2-hydroxyethyl)indol | 177,0 | 178,0 | 159,8 | 132,9 | 115,0 |
| 23,95 | 5-hydroxytryptophan | 220,0 | 221,0 | 204,0 | 161,9 | |
| 37,32 | 3-Carboxy-6-hydroxy-β-carbonyl | 228,0 | 229,0 | 210,0 | 183,0 | 101,1 |
| 33,09 | Hytioerectine B | 246,0 | 247,0 | 229,9 | 203,9 | 174,0 |
| 30,11 | Griffonine | 329,0 | 330,0 | 167,9 | | |
| 44,47 | Hyrtiosulawesine | 343,0 | 344,0 | 228,9 | 200,9 | 182,9 |
| | Peak X | 234,0 | 235,0 | 217,0 | 162,0 | 181,0 |
| | Peak E | 435,0 | | | | |

| | g | mL | FD |
|---|---|---|---|
| 95 | 0,0486 | 10 | 5 |
| 70 | 0,0467 | 10 | 5 |

Area

| # | RT | Compound | Area | | mg/mL | | mg/g as per Dilution Factor | | | | |
|---|---|---|---|---|---|---|---|---|---|---|---|
| | | | R1 | R2 | R1 | R2 | R1 | R2 | MEAN | ST. DEV. | %CV |
| n.d | 35,10 | 1H-indole-3-carboxylic acid | | | 0 | 0 | 0,00 | 0,00 | n.d. | | |
| n.d | 38,10 | 5-hydroxytryptamine | | | 0 | 0 | 0,00 | 0,00 | n.d. | | |
| n.d | 4,35 | 5-hydroxy-3-(2-hydroxyethyl)indol | | | 0 | 0 | 0,00 | 0,00 | n.d. | | |
| 13 | 35,95 | 5-hydroxytryptophan | 6,63E+09 | 6,12E+09 | 0,660043 | 0,609694 | 679,06 | 627,26 | **653,16** | 2,88 | 0,44 |
| n.d | 37,32 | 3-Carboxy-6-hydroxy-β-carbonyl | | | 0 | 0 | 0,00 | 0,00 | n.d. | | |
| 11 | 33,09 | Hytioerectine B | 31820170 | 30456871 | 0,003168 | 0,003032 | 3,26 | 3,12 | **3,19** | 0,12 | 3,76 |
| 17 | 32,99 | Griffonine | 7264692 | 71478540 | 0,000723 | 0,007116 | 0,74 | 7,32 | **4,03** | 0,06 | 1,49 |
| 20 | 39,01 | Hyrtiosulawesine | 9521621 | 9423698 | 0,000948 | 0,000938 | 0,98 | 0,97 | **0,97** | 0,01 | 1,03 |
| 14 | 36,01 | Peak X | 3,34E+08 | 3,1E+08 | 0,033226 | | 34,18 | | **34,18** | 0,01 | 0,03 |
| 19 | 37,98 | **Peak E** | 53820262 | 51248582 | 0,005358 | | 5,51 | | **5,51** | 0,01 | 0,18 |

| 5-HTTP to Alkaloid RATIO | | |
|---|---|---|
| | mg/g | |
| 5-hydroxytryptophan | 653 | |
| Other alkaloids | 48 | |
| Ratio | 14 :1 | |

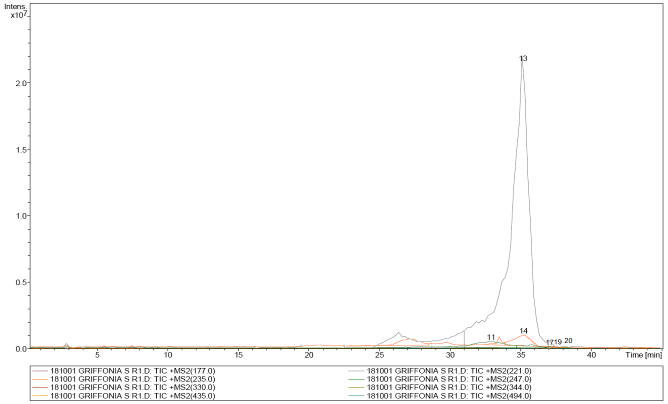


**The results show that the chemical characterization indicates that the real content of 5-HTTP is not 95%, but 65%.**
**The ratio between 5-HTTP and other alkaloids is 14:1**
**The presence of peak E indicates a possible bacterial fermentation process**
